# Locomotory Profile, Heart Rate Variability, and Blood Parameters Reveal Adaptive Responses in Endurance Horses Trained on Deep Sand

**DOI:** 10.3390/vetsci12111028

**Published:** 2025-10-23

**Authors:** Elisabetta Porzio, Samanta Mecocci, Giovanni Chillemi, Massimo Puccetti, Marco Pepe, Katia Cappelli, Francesca Beccati

**Affiliations:** 1 Department of Veterinary Medicine, University of Perugia, Via San Costanzo 4, 06126 Perugia, Italy; elisabettaporzio1@gmail.com (E.P.); samanta.mecocci@unipg.it (S.M.); marco.pepe@unipg.it (M.P.); francesca.beccati@unipg.it (F.B.); 2 Department for Innovation of Biological Systems, Agro-Food and Forest Systems (DIBAF), University of Tuscia, 01100 Viterbo, Italy; 3 Department of Experimental Medicine, University of Rome “Tor Vergata”, 00133 Rome, Italy; chillemi@med.uniroma2.it; 4 Dubai Equine Hospital, Zabeel, Dubai P.O. Box 9373, United Arab Emirates; drpuccetti@gmail.com; 5 Sport Horse Research Centre, Department of Veterinary Medicine, University of Perugia, Via San Costanzo 4, 06126 Perugia, Italy

**Keywords:** equine, athlete, exercise, autonomic regulation, fitness tracker

## Abstract

**Simple Summary:**

Endurance horses are special athletes that must sustain effort over very long distances, often in challenging conditions. In the United Arab Emirates, endurance horses are usually trained on deep sand, which is thought to be particularly demanding, as it requires high muscular effort compared to firm ground. This study investigated how endurance horses adapted to deep sand training by looking at their movement patterns, heart function, and blood changes. Results showed that horses mainly increased their stride length to reach higher speeds on sand, and that those with better recovery capacity had higher parasympathetic activity, a marker of fitness and fatigue resilience. Blood tests showed increases in red blood cell measures after training, indicating high oxygen-carrying capacity during exercise. These findings show that modern monitoring tools can help trainers and veterinarians track fitness, reduce the risk of fatigue or injury, and improve both performance and welfare in endurance horses.

**Abstract:**

Training on deep sand is commonly employed in endurance horses, but its physiological adaptation remains poorly characterized. This study aimed to characterize locomotor adaptations during a 7 km controlled-speed canter on deep sand in eighteen endurance horses, to identify heart rate variability (HRV) components, and to investigate changes in hematological variables before and after exercise. Stride frequency (SF) and stride length (SL), HRV, and hematological profiles were recorded during exercise and recovery with a fitness tracker. Associations between maximum speed and locomotor parameters were assessed by linear regression, while Pearson’s correlation assessed HRV relationships, also with physiological parameters. Hematological parameters were assessed with paired t-test before and after training. SL percentage change was the strongest predictor of speed (β = 0.677). HRV analysis revealed delayed parasympathetic reactivation; the parasympathetic recovery index (PNS REC) was correlated with mean RR interval on the ECG (r = 0.968) and heart rate (r = −0.964) during recovery. Post-exercise rectal temperature showed correlations with HRV recovery indices. Hematological evaluation revealed post-exercise increases in red blood cell count, hematocrit, hemoglobin, and corpuscular indices. SL plays a predominant role in achieving higher speeds on deep sand, while PNS REC emerges as a practical and accessible marker of autonomic recovery and fatigue. Horses with enhanced thermoregulation recover better. Hematological results confirm a physiological stress response that may optimize oxygen delivery. Integrating locomotor, cardiovascular, and hematological monitoring may improve management and welfare in endurance training.

## 1. Introduction

Endurance horses represent a unique model of the equine athlete, as they are required to sustain prolonged aerobic effort in the Federation Equestre Internationale (FEI) competitions over distances ranging from 100 to 160 km, often under challenging environmental conditions [1]. Performance in this discipline depends on a delicate balance between cardiovascular efficiency, metabolic resilience, thermoregulation, and musculoskeletal integrity [2,3]. Unlike speed-oriented equestrian disciplines, endurance riding places greater emphasis on fatigue resistance, recovery capacity, and long-term soundness [4]. For these features, endurance riding is one of the most energetically demanding equestrian discipline, making the evaluation of fitness essential. Training programs must be carefully designed to enhance aerobic capacity while minimizing the risk of overtraining and injury [5,6]. In this context, the standardization and objective monitoring of training sessions are of paramount importance. By continuously assessing locomotor patterns, heart rate dynamics, and autonomic regulation, trainers and veterinarians can better tailor conditioning strategies, identifying early signs of fatigue or maladaptation, and optimizing the welfare and competitive longevity of the endurance horse [7]. Traditionally, exercise capacity in horses is assessed using the heart rate (HR) response, which is influenced by the balance between sympathetic and parasympathetic efferent controls [8,9]. In human medicine, the more promising method to monitor individual adaptation to training involves the monitoring of the cardiac autonomic nervous system through the measurement of exercise [10] and recovery heart rate variability (HRV) [11]. Negative adaptation to training is thought to be associated with post-exercise sympathetic nervous system hyperactivity that may lead to ischemic heart disease, ventricular arrhythmias, and sudden cardiac death [12,13]. In the equestrian endurance discipline, rapid cardiac recovery is a key indicator of fitness and success and serves as a reliable marker of fatigue during competition [14], which highlights the importance of evaluating post-exercise parasympathetic reactivation by using vagal-related indices of HRV. In equines, cardiac activity is influenced by various factors, including individual level of training [15], age [14], duration of exercise [5], and environmental conditions [1], but the effect of track surface on cardiovascular physiology is unknown. Studies in human sports medicine demonstrated that training on soft and compliant surface, like deep sand, increases propulsive muscular effort, resulting in a higher HR at lower speed [16]. This would allow training of the cardiorespiratory system without reaching the maximal speed, decreasing stress on osteoarticular, tendinous, and ligamentous structures. In recent years, equine monitoring systems have undergone rapid technological development. The Equimetre^®^ 2.0 is a wearable device designed for monitoring the training and performance of horses. The device integrates surface ECG electrodes, inertial sensors (accelerometer and gyroscope), and multi-constellation GNSS to record heart rate, heart rate variability, stride parameters, and speed during field exercise. It introduced significant improvements, including real-time data transmission via 4G network, enhanced mechanical robustness, and a more compact design, thereby increasing reliability and expanding field applications [17]. At the same time, researchers have explored the use of inertial measurement units (IMU) to assess locomotor symmetry and enable early detection of lameness, often supported by artificial intelligence algorithms and convolutional neural networks, with promising diagnostic accuracy [18,19]. Other innovative approaches include the analysis of respiratory patterns during exercise using microphones combined with deep learning models, capable of identifying breathing dynamics with high precision [20]. These advances highlight how the integration of cardiovascular, respiratory, locomotor, and behavioral data may pave the way toward equine deep phenotyping, in which tools such as Equimetre^®^ are positioned within a broader ecosystem of digital technologies applied to performance monitoring and athlete welfare. Moreover, Equimetre^®^, has been validated to accurately record HR in conjunction with telemetric electrocardiogram (ECG) during training sessions, providing information on HRV [21]. Its accuracy for HR and HRV measurement, as well as for arrhythmia detection, has been validated against telemetric ECG recordings in controlled and field conditions [9,21]. This technology can monitor cardiovascular response to exercise and recovery, characteristic of locomotory function and speed. To date, no studies have been conducted to report such parameters for endurance horses trained in deep sand. Moreover, the respiratory system is the primary factor for oxygen (O_2_) delivery, aerobic metabolism, and athletic performance in the horse [22]; indeed, during exercise, ventilation must increase in response to the metabolic demand of exercising muscle. Since training in deep sand requires elevated muscular activity, and since during cantering there is a tight locomotor-respiratory coupling, locomotor adaptation to sand needs to be investigated. The aims of this study were: (i) to characterize the locomotor adaptations to deep sand by analyzing stride frequency (SF) and stride length (SL) during canter; (ii) to identify HRV components in endurance horses trained in deep sand; (iii) to investigate the changes in some hematological variables before and after training on deep sand. Hematological evaluation, indeed, provides complementary insight into the physiological adaptations to exercise. In equine athletes, complete blood count (CBC) is routinely performed to assess health and training status, as it provides information on splenic contraction, plasma volume shifts, and oxygen transport efficiency. In this study, these classical hematological parameters were analyzed because they are part of the standard CBC routinely used in athletic horses and allow non-invasive assessment of physiological adaptation. The innovative application is the integration of these hematological indicators with real-time physiological and locomotor data collected by a wearable fitness tracker. This approach enables a better interpretation of blood changes in the context of autonomic and mechanical workload and suggests that, in the future, continuous digital monitoring could complement or even replace routine hematological testing for evaluating training adaptation and fitness in endurance horses.

The authors hypothesized that the primary locomotor strategy for acceleration relies on increasing SL, a mechanism representing the first mechanism of acceleration in longer-distance performers, thereby contributing to performance efficiency. Furthermore, the authors proposed that superior training adaptation in endurance horses is reflected by greater HRV amplitude, indicating enhanced parasympathetic activity. Lastly, the authors hypothesized that a relatively short training session on deep sand could also result in changes in the hematological variables, resulting in mobilization of splenic erythrocytes and, therefore, increasing the oxygen transport capacity.

## 2. Materials and Methods

### 2.1. Horses

Eighteen elite endurance horses were included in this study. All horses were stabled in the same training center (Rabdan Endurance Stable, Dubai, United Arab Emirates) and were subjected to the same management in terms of training and nutrition. The study population included 11 Arabian and 7 Anglo-Arabian endurance horses, aged from 8 to 20 years (median 13 yo), consisting of 6 mares, 11 geldings, and 1 stallion. Information on each horse’s training and racing profile (such as categories raced, total distance covered during races, number of races, and weekly training schedules) was obtained from the FEI database or orally obtained by the trainer. The level of training of each horse was determined based on the highest Concours d’Endurance Internationale (CEI) category in which the animal had competed during the 2024–2025 season; 2 horses competed in CEI*, 11 horses in CEI**, and 5 in CEI***.

### 2.2. Training Session

Data were collected during a training session on deep sand in the Dubai desert. All horses underwent a dynamic examination at trot in straight line and a short clinical examination (HR, respiratory rate, rectal temperature) before the start of the training to assess that they were fit to perform. Before the beginning of the training session, each horse was equipped with a fitness tracker (Equimetre^®^, Arioneo, Paris, France) fitted as previously described [21]. Throughout all training sessions, the ambient temperature (°C) and humidity (%) were recorded using a portable device (GPS Temperature and Humidity Data Logger GSP-6, Trans Instruments Ltd., Leicester, UK) held within 4 km from the deep sand training surface. The training exercise consisted of 15 km total distance. The warm-up was performed on compact sand and included 2 km of walk, followed by 2 km of trot at unregulated speed. This was followed by 7 km of canter on an oval deep-sand track at a controlled speed of 21 km/h (5.8 m/s), regulated using a GPS monitoring device (Garmin^®^ Forerunner 945 watch, Garmin Ltd., Olathe, KS, USA) held by the rider. The return to the stables was performed on compact sand and included 2 km of trot at unregulated speed and 2 km of canter at unregulated speed. Upon arrival, rectal temperature was collected and horses were unsaddled, cooled down with cold water, and underwent a dynamic examination at trot in straight line to assess for any signs of lameness following the training. Data recorded from the Equimetre^®^ were downloaded via a wireless connection and automatically uploaded to the online platform for analysis.

### 2.3. Equimetre^®^ Data Analysis

**Figure 1:**
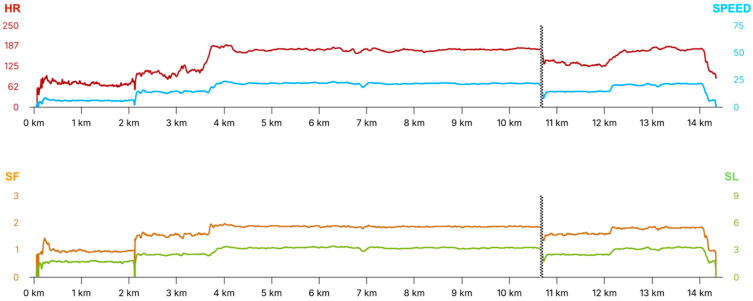
Example of Equimetre^®^ physiological and biomechanical parameters recorded during the training session. The upper panel shows the heart rate (HR) (red line, bpm; left *y*-axis) and speed (blue line, km/h; right y-axis) as a function of distance (*x*-axis, km). HR increases progressively after the initial warm-up, stabilizes during steady-state running on deep sand, and decreases toward the end of the session. Speed follows a similar pattern. The lower panel displays stride frequency (SF) (orange line, stride/s; left *y*-axis) and stride length (SL) (green line, m; right *y*-axis) as a function of distance (*x*-axis, km). Both parameters show initial fluctuations during the warm-up, stabilization during the main running phase, and a decline toward the end of the session. The dotted vertical line at ~11 km indicates the end of the 7 km canter on deep sand.

Data on locomotor performance during the 7 km of canter on deep sand (HIGH_EXERCISE) and data on cardiac activity during HIGH_EXERCISE and during the first 5 min of 2 km recovery trot (REC_EXERCISE) were collected using Equimetre^®^. Speed- and stride-related variables were obtained by a combination of GNSS (GPS + GLONASS + Galileo), which provided real-time positioning data used to calculate velocity, and accelerometers and gyroscopes which measured the acceleration patterns of the horse’s movement. From inertial sensors, SF and SL were derived. HR data were measured using surface electrodes embedded in the device’s girth, which captured the ECG signal throughout the session (Figure 1). All raw and processed parameters were automatically transmitted via Bluetooth and cellular connection to the Equimetre^®^ cloud-based platform immediately after exercise. The platform stored the data in the individual horse’s profile, where they were exported through the online dashboard for analysis. Locomotor parameters included maximal speed (Smax), stride frequency (SFmax) and stride length (SLmax) at maximal speed, and stride frequency (SF20) and stride length (SL20) at 20 km/h. Cardiac parameters considered for HIGH_EXERCISE and REC_EXERCISE included mean, maximum, and minimum HR. ECG data collected by the device during HIGH_EXERCISE and REC_EXERCISE were exported as csv files from the online dashboard and imported into Kubios^®^ HRV Standard (Kubios HRV software [version 3.0.2], Biomedical Signal Analysis Group, Department of Applied Physics, University of Kuopio, Finland) for analysis of time-domain variables to determine HRV. An automatic identification of the RR was performed with a strong artifact correction filter applied to reduce error across the sample [23]. Occasional ectopic beats were visually identified and manually replaced with interpolated adjacent RR interval values. The mean RR interval, standard deviation of the normal RR (SDNN), and root mean square of the standard deviation (RMSSD) were calculated for HIGH_EXERCISE and REC_EXERCISE. Poincaré plot analysis identified the shape of the ellipse made by plotting each RR interval as a function of the previous RR interval. The ellipse’s width (SD1) and length (SD2), together with parasympathetic (PNS) and sympathetic (SNS) indices, were collected during HIGH_EXERCISE and REC_EXERCISE. All the data were exported in text file for statistical analysis.

### 2.4. Blood Parameters

Blood samples were collected by the attending stable veterinarian via venipuncture of the external jugular vein, using a vacutainer system with EDTA collection tubes. These samples were obtained as part of routine clinical monitoring to assess the horses’ health status during training and ensure their fitness prior to training. The complete blood count, including red and white cell parameters, was therefore performed for clinical purposes. The data were subsequently analyzed for research purposes to investigate hematological changes associated with exercise on deep sand.

### 2.5. Statistical Analyses

Data from Equimetre^®^ were analyzed using JASP free statistical software (version 0.18.3, JASP Team, Amsterdam, The Netherlands). Numerical variables were presented as mean ± standard deviation or median and range, as appropriate; nominal data were presented as frequencies and percentages. Descriptive statistic was performed for rectal temperature before and after exercise, and environmental conditions; in addition, descriptive statistic was performed for blood analysis results, locomotor parameters, and HRV analysis stratified by HIGH_EXERCISE and REC_EXERCISE. Numerical data were assessed for homoscedasticity using Shapiro–Wilk test, for normality, and Levene test, for homogeneity of the variance, and tests were applied as appropriate. Pearson’s correlation coefficients were calculated among Smax, SFmax, SLmax, SF20, and SL20. For each horse, the difference between the measurements at maximum velocity and those at 20 km/h was calculated as ΔSF and ΔSL. The percentage variation was calculated as:

Δ%SF = ((SFmax − SF20)/SF20)) × 100

Δ%SL = ((SLmax − SL20)/SL20) × 100

A linear regression model was built to estimate the relationship between Smax and percentage of changes (Δ%SF and Δ%SL). Regression coefficients (standardized) were interpreted to assess the relative contribution of each variable to maximum speed. To assess relationships among all HRV variables and physiological parameters, Pearson’s correlation was performed. Correlation coefficients were interpreted according to thresholds previously described in the literature, where the strength of the relationship is classified as poor (r < 0.20), weak (0.20 ≤ r < 0.50), moderate (0.51 ≤ r < 0.80), strong (0.81 ≤ r < 0.99), and perfect (r ≥ 0.99) [24]. Hematological parameters were measured in horses before (PRE) and after (POST) training session, and paired-sample *t*-tests were performed for each parameter to compare PRE and POST values. The statistical analyses and boxplots were performed in R, version 4.3.0 (R Core Team, Vienna, Austria) using the tidyverse 2.0.0, ggplot2 3.5.2, and ggpubr 0.6.1 packages. Significance was set at *p* < 0.05.

Given the limited number of elite endurance horses available, no a priori sample size calculation was performed. Instead, a post hoc sensitivity analysis was conducted (α = 0.05, power = 0.80) to estimate the minimum detectable effect sizes with the available sample (*N* = 18) and presented in Supplementary Table S1. This sensitivity analysis indicated that the study was powered to detect medium-to-large effects in within-horse and correlational analyses.

## 3. Results

### 3.1. Horses and Environment

All horses were sound at the end of the training session. Rectal temperatures were homoscedastic; environment data were not normally distributed. Rectal temperature before the training session was 37.0 ± 0.30 °C; at the end, it was 39.8 ± 0.9 °C. All horses exercised at relatively cool temperatures (15.22 ± 3.92 °C), and relative humidity was 67.88 ± 18.45%. All horses were trained under safe environmental conditions, with a Heat Index < 130.

### 3.2. Locomotor Parameters

Locomotors parameters were normally distributed. SF20 and SL20 were 1.77 ± 0.05 strides/s and 3.14 ± 0.08 m/stride, respectively; Smax, SFmax, and SLmax were 22.8 ± 1.3 km/h, 1.85 ± 0.05 strides/s and 3.44 ± 0.17 m/stride, respectively. A moderate-to-strong negative correlation was found between SF20 and SL20 (r = −0.99), indicating that as SF increased, SL decreased. A moderate-to-strong positive correlation was found between SFmax and SF20 (r = 0.78) and between SLmax and SL20 (r = 0.65), indicating that both SF and SL increased between 20 km/h and maximum speed; however, the percentage change was greater for Δ%SL (9.76% ± 4.10) than Δ%SF (4.43% ± 1.83) (*p* < 0.001). Percentage change in SL was the strongest predictor of maximum speed (β = 0.677, *p* < 0.001), indicating that horses exhibiting a greater increase in SL between 20 km/h and maximum speed achieved higher speeds.

### 3.3. HR and HRV

HRV parameters were homoscedastic. Descriptive analysis is reported in Table 1. Results indicated wide inter-individual variability, with overall similar distributions across groups. A strong correlation was observed between PNS REC and MEAN RR REC (r = 0.968, *p* < 0.001), as well as between PNS REC and MEAN HR REC (r = −0.964, *p* < 0.001), indicating that horses with better recovery capacity exhibited the highest PNS REC values. Moderate-to-strong correlation was found between post-exercise rectal temperature and mean RR REC (r = −0.487; *p* = 0.041), mean HR REC (r = 0.495; *p* = 0.037), and PNS REC (r = −0.480; *p* = 0.044), suggesting that horses with greater thermoregulatory capacity exhibit more efficient recovery.

### 3.4. Blood Analysis

**Figure 2:**
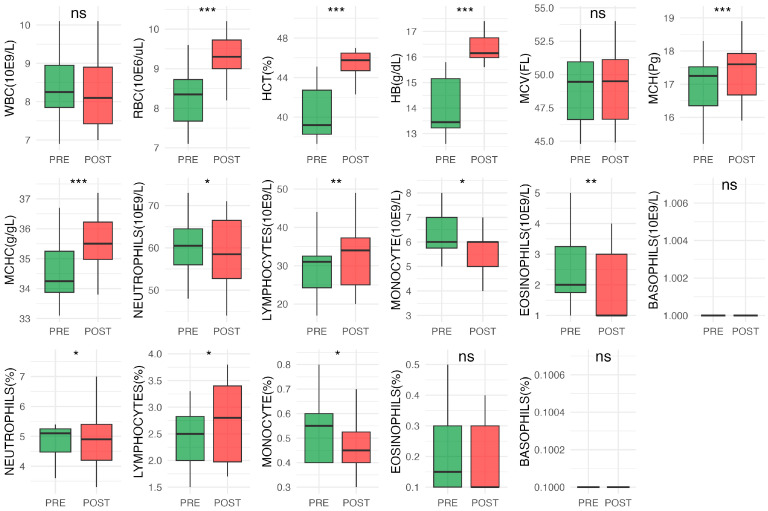
Boxplots for each hematological parameter, displaying PRE (green) and POST values (red). Significant difference of the PRE–POST comparisons was annotated directly on the figures using asterisks (* *p* < 0.05, ** *p* < 0.01, *** *p* < 0.001). ns = not significant.

For hematological parameters, a significant increase after the training was observed for hematocrit (HCT), red blood cell count (RBC), hemoglobin (HB), mean corpuscular hemoglobin (MCH), and mean corpuscular hemoglobin concentration (MCHC). Despite white blood cells (WBC) being stable, a slight increase in lymphocytes and a decrease in neutrophils, monocytes, and eosinophils were observed after training (Figure 2 and Table S2).

## 4. Discussion

### 4.1. Locomotion

The first aim of this study was to investigate the locomotor strategy of endurance horses trained on deep sand. In human sports medicine, sand training enhances physiological conditioning, requiring nearly double the energy expenditure compared to firm ground, due to resistance to forward movement and increased muscular effort to prevent slippage [16]. For equine athletes, optimizing locomotion on sand involves both mechanical adjustments and metabolic adaptations. During energetically demanding training, success largely depends on the individual’s aerobic capacity and ability to maintain low oxygen consumption. Evaluating the locomotor strategy is therefore essential, as individuals with better movement economy require less oxygen [25]. Peak performance in marathon runners is typically achieved by optimizing SL while maintaining high aerobic capacity and resistance to fatigue [16]. Results support the hypothesis that increasing SL is the primary strategy used by endurance horses to accelerate and reach higher speeds. Previous research has linked running economy—defined as the amount of oxygen consumed per unit of distance—to coordination between respiratory patterns and gait in Arabian horses [26]. During cantering, stride and respiratory rates are synchronized through locomotor-respiratory coupling, which reduces the oxygen cost of breathing [2]. Interestingly, horses can regulate tidal volume independently of SL [27]. This suggests that horses may increase tidal volume in response to metabolic demands unrelated to speed, as could be the case during training on deep sand. Human studies indicate that running on sand significantly increases energy expenditure while the shock-absorbing properties of sand may reduce impact forces during activity, potentially lowering the risk of muscle damage and soreness [28]. In contrast, one equine study reported a high incidence of pelvic fatigue fractures during deep-sand training [29]. Further research is needed to determine whether steady-rate training on deep sand can improve movement economy, reduce oxygen consumption, and ultimately improve performance in endurance horses. In this study, horses showing a greater percentage increase in SL between 20 km/h and peak speed achieved higher maximum speeds, suggesting SL as a potential performance indicator. However, as horses were trained at a steady-state speed of 21 km/h and only briefly exceeded this pace, the measured peak speeds may not reflect true maximal capacity. Additionally, stride frequency becomes increasingly important at higher speeds [16], leaving the predictive value of SL uncertain. Given its moderate heritability in Thoroughbreds [30], SL may also hold potential for selection programs.

### 4.2. HRV

The second aim of this study was to evaluate the HRV during deep sand training and recovery. In human athletes, HRV monitoring devices are routinely used to assess physiological responses to workload, to prevent overreaching or overtraining, and to provide insights in cases of poor performance [16]. In horses, HRV reveals vagal reactivation post-exercise, an important marker of endurance capacity [14]. In this study, post-exercise recovery (REC_EXERCISE) was characterized by increased vagal-related indices (RR interval, SDNN, PN index, SD2) and decreased sympathetic indices (SNS index, SD1) compared with high-intensity exercise (HIGH_EXERCISE). RMSSD remained unchanged, and HR stayed elevated during recovery. In humans, RMSSD and SD2 are considered markers of recovery capacity and adaptability to stress [31,32]. Similar findings have been reported in endurance horses, where lower HR recovery has been associated with higher RMSSD recovery [14]. The persistently high HR and low RMSSD during recovery observed in this study suggest delayed vagal reactivation, likely due to the high metabolic load of deep-sand training. This may also reflect that recovery data were collected during 5 min trotting, rather than during walking and cooling-down, as typically occurs in endurance competitions. Among the HRV indices, only SD2 (long-term RR interval variability) increased during REC_EXERCISE. Interestingly, horses with superior recovery capacity, characterized by lower HR and higher RR intervals post-exercise, exhibited higher PNS index. Since this index reflects parasympathetic activity, it may serve as a practical and easily obtainable marker of training adaptation, supporting current evidence linking enhanced vagal activity to improved fitness and performance [33].

Environmental factors were recorded and investigated during this study, including temperature and humidity; these factors influence cardiorespiratory responses during both exercise and recovery, increasing the risk of metabolic failure in endurance competitions via mechanisms such as electrolyte depletion and impaired thermoregulation [34]. In the present study, the influence of environmental conditions HRV components were not evaluated because of the ethical limitations (heat index below 130, recommended by the American Association of Equine Practitioners Heat Index guidelines). Interestingly, horses that exhibited elevated post-exercise body temperature demonstrated impaired cardiac recovery, including reduced RR interval variability, higher HR during recovery, and lower PNS recovery values. This may suggest that individuals with diminished thermoregulatory capacity recover less efficiently and emphasize the potential of HRV monitoring to detect early signs of exhaustion due to heat accumulation.

### 4.3. Hematological Adaptation

These findings from HRV analysis are further supported by hematological results, which provide additional evidence of the physiological adaptations triggered by deep sand training. In particular, RBC, HCT, and Hb levels increased after exercise, suggesting post-exercise hemoconcentration. This effect has been widely described in both equine and human athletes as a consequence of splenic contraction and plasma volume shifts, enhancing oxygen transport capacity during sustained exertion [35]. Moreover, in human athletes, splenic contraction has been shown to trigger hemoconcentration in a dose-dependent manner with increasing exercise intensity [36], supporting the idea that similar mechanisms may act in equine athletes under high metabolic demand. Regarding red blood cell indices, both MCH and MCHC significantly increased after exercise, suggesting an improved oxygen-carrying capacity per erythrocyte. These results indicate that, in addition to hemoconcentration, deep sand training may enhance hemoglobin concentration within red blood cells, thus optimizing oxygen transport under strenuous conditions [37]. Similar increases in MCHC have been reported in Thoroughbred racehorses during maximal exertion, reinforcing its role as a sensitive marker of exercise-induced erythrocytic adaptation [34]. With respect to leukocyte populations, we observed a significant redistribution of white blood cell subsets, characterized by a post-exercise decrease in lymphocyte, eosinophil, and monocyte counts, and an increase in neutrophils. These shifts mirror findings from recent endurance race studies in horses [38] and are consistent with known stress- and catecholamine-mediated leukocyte trafficking [39]. Notably, similar patterns of neutrophil functional suppression and acute leukocyte modulation occur immediately post-race in Thoroughbreds [40], in line with an acute phase immune adaptation rather than inflammatory pathology. Overall, the hematological results highlight the acute physiological adjustments that enable endurance horses to sustain energetically demanding work on deep sand.

### 4.4. Limitations

Several limitations should be considered when interpreting the findings of the present study. Firstly, the highly demanding nature of deep-sand training and the absence of a proper cool-down phase may have affected the HRV recovery data, limiting their value as precise indicators of post-exercise parasympathetic activation. Second, although training occurred under controlled environmental conditions (Heat Index < 130), the potential impact of temperature and humidity fluctuations on cardiac responses was not assessed. To strengthen the applicability of HRV monitoring in endurance training, future studies should include larger and more heterogeneous populations and standardized recovery protocols.

## 5. Conclusions

This study provides new insights into the physiological adaptations of endurance horses trained on deep sand, highlighting the combined importance of locomotor strategy, cardiovascular parameters, and hematological response. The findings highlight that SL plays a predominant role in achieving higher speeds during canter on deep sand, suggesting its potential as a performance-related parameter. Additionally, HRV analysis identified the parasympathetic recovery index (PNS REC) as a promising, easily obtainable parameter to monitor autonomic recovery and fatigue status. The significant post-exercise changes observed in hematological data further support the hypothesis that deep sand training elicits controlled physiological stress, contributing to enhanced oxygen transport capacity. Overall, the integration of locomotor, cardiovascular, and hematological data supports the use of digital tools such as fitness trackers for performance monitoring in endurance horses. The use of a fitness tracker to assess the daily adaptation to training is a concept largely investigated in human sports medicine, where training programs based on monitoring HRV seem to increase performance in human endurance athletes [11]. The findings of this study support the potential value of similar approaches in equine endurance, with applications for both performance optimization and welfare monitoring.

## Figures and Tables

**Table 1:** HRV parameters (mean ± SD) during the 7 km canter on deep sand training (HIGH_EXERCISE) and during 5 min trot recovery (REC_EXERCISE).

HRV Parameters	HIGH_EXERCISE Mean ± SD	REC_EXERCISE Mean ± SD	ΔHIGH-REC %
MEAN RR ms	365 ± 21	464 ± 29	27 ± 11
MEAN HR beats/min	165 ± 9	130 ± 8	−21 ± 7
HR MIN beats/min	129 ± 13	113 ± 14	−12 ± 13
HR MAX beats/min	180 ± 10	173 ± 12	−4 ± 9
SDNN ms	4 ± 1	6 ± 2	64 ± 67
RMSSD ms	4 ± 2	4 ± 1	−10 ± 44
PNS	−4 ± 0.1	−4 ± 0.2	−17 ± 6
SNS	16 ± 2.848	12 ± 3	−25 ± 23
SD1%	44 ± 7	27 ± 8	−37 ± 22
SD2%	56 ± 9	73 ± 8	29 ± 24

## Data Availability

The original contributions presented in this study are included in the article/Supplementary Materials. Further inquiries can be directed to the corresponding author.

## Supplementary Materials

The following supporting information can be downloaded at: https://www.mdpi.com/article/10.3390/vetsci12111028/s1, Table S1: Sensitivity analysis performed post hoc on the statistical tests; Table S2: Hematological (mean ± SD) parameters measured before (PRE) and after (POST) deep sand training exercise.

## References

[1] Nagy A., Murray J.K., Dyson S.J. (2014). Horse-, rider-, venue- and environment-related risk factors for elimination from Fédération Equestre Internationale endurance rides due to lameness and metabolic reasons. Equine Vet. J..

[2] Hinchcliff K.W., Kaneps A.J., Geo R.J., Van Erck-Westergren E. (2025). Equine Sports Medicine and Surgery: Basic and Clinical Sciences of the Equine Athlete.

[3] Robert C., Goachet A.-G., Fraipont A., Votion D.-M., Van Erck-Westergren E., Leclerc J.-L. (2010). Hydration and electrolyte balance in horses during an endurance season. Equine Vet. J. Suppl..

[4] Marlin D., Nankervis K.J. (2002). Equine Exercise Physiology.

[5] Cottin F., Barrey E., Lopes P., Billat V. (2006). Effect of repeated exercise and recovery on heart rate variability in elite trotting horses during high intensity interval training. Equine Vet. J. Suppl..

[6] Munsters C., Siegers E., Sloet van Oldruitenborgh-Oosterbaan M. (2024). Effect of a 14-Day Period of Heat Acclimation on Horses Using Heated Indoor Arenas in Preparation for Tokyo Olympic Games. Animals.

[7] Paris A., Accorroni L., Pepe M., Cappelli K., Chiaradia E., Mecocci S., Tognoloni A., Passamonti F., Pilati N., Cercone M. (2024). Retrospective study of standardised field exercise test on injury development, blood lactate and recovery time in endurance horses. Comp. Exerc. Physiol..

[8] Kapteijn C.M., Frippiat T., van Beckhoven C., van Lith H.A., Endenburg N., Vermetten E., Rodenburg T.B. (2022). Measuring heart rate variability using a heart rate monitor in horses (*Equus caballus*) during groundwork. Front. Vet. Sci..

[9] ter Woort F., Dubois G., Didier M., Van Erck-Westergren E. (2021). Validation of an equine fitness tracker: Heart rate and heart rate variability. Equine Vet. J..

[10] Sandercock G.R.H., Brodie D.A. (2006). The use of heart rate variability measures to assess autonomic control during exercise. Scand. J. Med. Sci. Sports.

[11] Plews D.J., Laursen P.B., Stanley J., Kilding A.E., Buchheit M. (2013). Training adaptation and heart rate variability in elite endurance athletes: Opening the door to effective monitoring. Sports Med..

[12] Brenner I.K., Thomas S., Shephard R.J. (1998). Autonomic regulation of the circulation during exercise and heat exposure. Inferences from heart rate variability. Sports Med..

[13] Zhang D.Y., Anderson A.S. (2014). The Sympathetic Nervous System and Heart Failure. Cardiol. Clin..

[14] Younes M., Robert C., Barrey E., Cottin F. (2016). Effects of Age, Exercise Duration, and Test Conditions on Heart Rate Variability in Young Endurance Horses. Front. Physiol..

[15] Bitschnau C., Wiestner T., Trachsel D.S., Auer J.A., Weishaupt M.A. (2010). Performance parameters and post exercise heart rate recovery in Warmblood sports horses of different performance levels. Equine Vet. J. Suppl..

[16] McArdle W.D., Katch F.I., Katch V.L. (2015). Exercise Physiology: Nutrition, Energy, and Human Performance.

[17] Equimetre. https://training.arioneo.com/en/equimetre-2-0-our-sensors-big-changes/.

[18] Calle-González N., Lo Feudo C.M., Ferrucci F., Requena F., Stucchi L., Muñoz A. (2024). Objective Assessment of Equine Locomotor Symmetry Using an Inertial Sensor System and Artificial Intelligence: A Comparative Study. Animals.

[19] Savoini B., Bertolaccini J., Montavon S., Deriaz M. (2025). Convolutional neural network for early detection of lameness and irregularity in horses using an IMU sensor. arXiv.

[20] Parmentier J.I.M., Aarts R.M., Hernlund E., Rhodin M., van der Zwaag B.J. (2025). Detecting and measuring respiratory events in horses during exercise with a microphone: Deep learning vs. standard signal processing. arXiv.

[21] ter Woort F., Dubois G., Didier M., Van Erck-Westergren E. (2022). Validation of an equine fitness tracker: ECG quality and arrhythmia detection. Equine Vet. J..

[22] Franklin S.H., Van Erck-Westergren E., Bayly W.M. (2012). Respiratory responses to exercise in the horse. Equine Vet. J..

[23] van Vollenhoven E., Fletcher L., Page P.C., Ganswindt A., Grant C.C. (2017). Heart Rate Variability in Healthy, Adult Pony Mares During Transrectal Palpation of the Reproductive Tract by Veterinary Students. J. Equine Vet. Sci..

[24] Akoglu H. (2018). User’s guide to correlation coefficients. Turk. J. Emerg. Med..

[25] O’Halloran J., Hamill J., McDermott W.J., Remelius J.G., Van Emmerik R.E.A. (2012). Locomotor-respiratory coupling patterns and oxygen consumption during walking above and below preferred stride frequency. Eur. J. Appl. Physiol..

[26] Cottin F., Metayer N., Goachet A.G., Julliand V., Slawinski J., Billat V., Barrey E. (2010). Oxygen consumption and gait variables of Arabian endurance horses measured during a field exercise test. Equine Vet. J. Suppl..

[27] Butler P.J., Woakes A.J., Anderson L.S., Roberts C.A., Marlin D.J. (1993). Stride length and respiratory tidal volume in exercising thoroughbred horses. Respir. Physiol..

[28] Binnie M.J., Dawson B., Pinnington H., Landers G., Peeling P. (2014). Sand training: A review of current research and practical applications. J. Sports Sci..

[29] Puccetti M., Beccati F., Denoix J.-M. (2022). Bone stress injuries and fatigue fractures of the pelvis in endurance horses. Equine Vet. J..

[30] Schrurs C., Blott S., Dubois G., Van Erck-Westergren E., Gardner D.S. (2022). Locomotory Profiles in Thoroughbreds: Peak Stride Length and Frequency in Training and Association with Race Outcomes. Animals.

[31] Hautala A., Tulppo M.P., Mäkikallio T.H., Laukkanen R., Nissilä S., Huikuri H.V. (2001). Changes in cardiac autonomic regulation after prolonged maximal exercise. Clin. Physiol..

[32] Goldberger J.J., Challapalli S., Tung R., Parker M.A., Kadish A.H. (2001). Relationship of heart rate variability to parasympathetic effect. Circulation.

[33] Mourot L., Bouhaddi M., Tordi N., Rouillon J.-D., Regnard J. (2004). Short- and long-term effects of a single bout of exercise on heart rate variability: Comparison between constant and interval training exercises. Eur. J. Appl. Physiol..

[34] Hinchcliff K.W., Kaneps A.J., Geor R.J. (2008). Equine Exercise Physiology: The Science of Exercise in the Athletic Horse.

[35] de Siqueira R.F., Fernandes W.R. (2024). Dynamic Hematological Responses in Endurance Horses: Unraveling Blood Physiological Markers of Exercise Stress and Recovery. Int. J. Equine Sci..

[36] Lindblom H., Pernett F., Schagatay E., Holmström P. (2024). Effect of exercise intensity and apnea on splenic contraction and hemoglobin increase in well-trained cross-country skiers. Eur. J. Appl. Physiol..

[37] Hodgson D.R., McKeever K.H., McGowan C.M. (2014). The Athletic Horse: Principles and Practice of Equine Sports Medicine.

[38] Vlaeva R., Sabev S., Ivanova Z. (2023). Hematological parameters in endurance horses pre and post 120 km race. Rev. Ciênc. Agroveterinár..

[39] Witkowska-Piłaszewicz O., Malin K., Dąbrowska I., Grzędzicka J., Ostaszewski P., Carter C. (2024). Immunology of Physical Exercise: Is Equus caballus an Appropriate Animal Model for Human Athletes?. Int. J. Mol. Sci..

[40] Zandoná Meleiro M.C., de Carvalho H.J.C., Ribeiro R.R., da Silva M.D., Salles Gomes C.M., Miglino M.A., de Santis Prada I.L. (2022). Immune Functions Alterations Due to Racing Stress in Thoroughbred Horses. Animals.

