# Supplementary material for: Locomotory Profile, Heart Rate Variability, and Blood Parameters Reveal Adaptive Responses in Endurance Horses Trained on Deep Sand
Source: Vet Sci. 2025 Oct 23;12(11):1028. doi: 10.3390/vetsci12111028 (PMC12656824; doi:10.3390/vetsci12111028)
Supplement: Supplementary file 1 [file vetsci-12-01028-s001.zip › vetsci-3900528-supplementary.pdf]

Supplementary Materials:

Table S1. Sensitivity analysis performed *post hoc* on the statistical tests applied on the study group considering 80% of power and a two-sided criterion for detection that allows for a maximum type I error rate of α = 0.05. Interpretation follows Cohen's conventional thresholds (small, medium, large).

Statistical test	***Comparison/ Parameter***	***Minimum detectable effect size***	***Interpretation***
Linear regression	Locomotor parameters	Cohen's f^2 = 0.41	≥95% Almost surely detect
Correlation	HRV, physiological and locomotor parameters	r = 0.66	≥95% Almost surely detect
Paired t-test	PRE vs. POST (Hematology)	Cohen's $\lvert\delta\rvert$ = 0.62	80-95% Probably detected

Table S2. Hematological (mean ± SD) parameters measured before (PRE) and after (POST) deep sand training exercise.

Parameters	*PRE mean ± SD*	*POST mean ± SD*	*Mean difference (POST vs PRE)*	*p-value*
WBC (x10^9/L)	8.42 ± 1.01	8.30 ± 1.12	-0.12	0.552
RBC (x10^{12}/L)	8.28 ± 0.76	9.32 ± 0.54	1.04	<0.001
HCT (%)	40.36 ± 2.63	45.63 ± 1.85	5.27	<0.001
HB (g/dL)	13.98 ± 1.11	16.20 ± 0.79	2.22	<0.001
MCV (FL)	48.93 ± 2.68	49.09 ± 2.83	0.16	0.16
MCH (Pg)	16.95 ± 0.97	17.42 ± 0.87	0.47	<0.001
MCHC (g/dL)	34.64 ± 1.06	35.51 ± 0.97	0.87	<0.001
NEUTROPHILS (x10^9/L)	60.83 ± 7.59	58.92 ± 8.59	-1.92	0.04
LYMPHOCYTES (x10^9/L)	29.42 ± 8.26	32.75 ± 8.88	3.33	0.001
MONOCYTES (x10^9/L)	6.17 ± 0.94	5.50 ± 1.17	-0.67	0.02
EOSINOPHILS (x10^9/L)	2.58 ± 1.44	1.92 ± 1.24	-0.67	0.004
BASOPHILS (x10^9/L)	1.00 ± 0.00	1.00 ± 0.00	0	NA
NEUTROPHILS (%)	5.15 ± 1.10	4.89 ± 1.03	-0.26	0.03
LYMPHOCYTES (%)	2.44 ± 0.63	2.71 ± 0.76	0.27	0.03
MONOCYTES (%)	0.53 ± 0.12	0.47 ± 0.12	-0.07	0.02
EOSINOPHILS (%)	0.22	0.18	-0.03	0.1
BASOPHILS (%)	0.10	0.10	0.00	NA
